# The Impact of Mechanically-Imposed Shear on Clogging, Fouling and Energy Demand for an Immersed Membrane Bioreactor

**DOI:** 10.3390/membranes8040104

**Published:** 2018-11-10

**Authors:** Simon Judd, Albert Odai, Pompilia Buzatu, Hazim Qiblawey

**Affiliations:** 1 Gas Processing Center, Qatar University, Doha, Qatar; albert.odai@qu.edu.qa (A.O.); pompilia.buzatu@qu.edu.qa (P.B.); 2 Cranfield Water Science Institute, Cranfield Univeristy, Cranfield, Bedford MK43 0AL, UK; 3 Department of Chemical Engineering, Qatar University, Doha, Qatar; hazim@qu.edu.qa

**Keywords:** membrane bioreactor, fouling, clogging, mechanical shear, specific energy demand

## Abstract

The impact of the application of mechanically-imposed shear on the propensity for fouling and clogging (or “sludging”—the agglomeration of sludge solids in the membrane channel) of an immersed flat sheet (iFS) membrane bioreactor (MBR) was studied. The bench-scale test cell used contained a single flat sheet fitted with a crank and motor to allow the membrane to be oscillated (or reciprocated) vertically at a low rate (20 RPM). The membrane was challenged with sludge samples from a local MBR installation treating petroleum industry effluent, the sludge having previously been demonstrated as having a high sludging propensity. Sludging was measured by direct visual observation of membrane surface occlusion by the agglomerated solids, with fouling being notionally represented by the rate of transmembrane pressure increase. Results demonstrated membrane reciprocation to have a more beneficial impact on sludging amelioration than on suppressing fouling. Compared with the stationary membrane, sludging was reduced by an average of 45% compared with only 13% for fouling suppression at the reference flux of 15 L·m^−2^·h^−1^ applied. The specific energy demand of the mechanical shear application was calculated as being around 0.0081 kWh·m^−3^, significantly lower than values reported from a recent pilot scale study on a reciprocated immersed hollow fibre MBR. Whilst results appear promising in terms of energy efficiency, it is likely that the mechanical complexity of applying membrane movement would limit the practical application to low flows, and a correspondingly small number of membrane modules.

## 1. Introduction

The principle of employing mechanical, rather than aeration-imposed, shear to sustain the flux of an immersed hollow fibre (iHF) membrane was reported more than 12 years ago [1,2]. These studies demonstrated the improvement in flux from applying a mechanical shear to immersed systems by vibrating (or oscillating or “reciprocating”) the iHF membrane, in much the same way as the more extensively reported and reviewed rotating and vibrating membrane disc systems [3,4].

**Figure 1:**
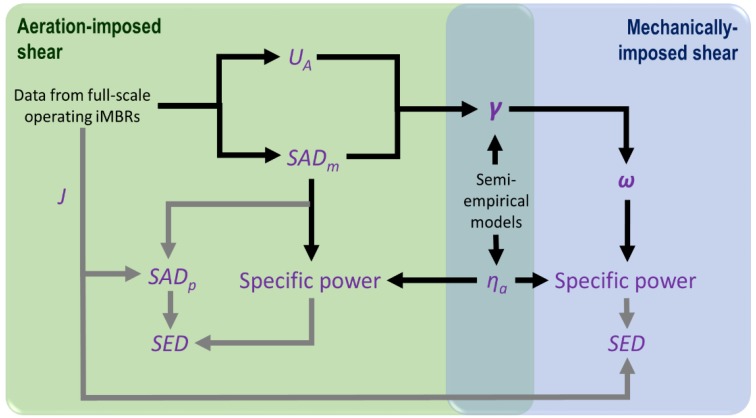
Determination of specific energy demand (SED) for mechanical-induced shear systems.

A critical factor in determining the efficacy of applying mechanical shear, is the ratio of the specific power demand $\bar{P^{\prime }}$, in kW per m^−2^ membrane area, with the shear rate *γ* in s^−1^. The shear may be generated either mechanically, through reciprocation of the membrane at some rate, or through air scouring at some specific aeration demand *SAD_m_*, in Nm^3^·h^−1^·m^−2^ (Figure 1), as in classical immersed membrane bioreactors (iMBRs). The applied shear then itself generates a flux *J* in m^3^·m^−2^·h^−1^, the $\bar{P^{\prime }}$/*J* ratio equating to the key normalized energy parameter of specific energy demand (SED) in kWh·m^−3^.

The shear-to-power ratio can be determined from first principles in the case of the mechanical system, as recently reported for a flat sheet (FS) membrane for which the comparative simplicity of the parallel flow channel geometry permits an analytical solution [5]. The same parameter for an aerated system is less easily determined because of the long-recognized complexity and dynamic nature of the shear generated by a moving air bubble [6], though the averaged value can be estimated [7]. Notwithstanding this, most recently reported pilot-scale studies of a mechanically shear-induced iHF [8,9] have demonstrated, through practical experimentation, the energy benefit of the mechanical system. Ho et al. [8] have reported an energy reduction of ~42% over that required for applying the same shear by conventional aeration. This figure is within the broad range of a 20% to 70% specific power reduction calculated for an FS geometry by Buzatu et al. [5], the energy saving increasing with decreasing shear (i.e., at lower reciprocation or aeration rates).

Whilst reported outcomes thus far appear encouraging, there are a number of key assumptions made in the mathematical development. Firstly, assumptions concerning the mixed liquor rheological properties significantly impact on the $\bar{P^{\prime }}$: *γ* relationship. Secondly, it is assumed that the *J*: *γ* relationship is independent of the method of the shear generation. Lastly, there has been no direct comparison between the two immersed membrane configurations (i.e., HF and FS) with reference to either the $\bar{P^{\prime }}$ or SED relationships, either from mathematical modeling or practical experimentation.

The current paper attempts to quantify the SED of a purely mechanical immersed flat sheet (iFS) MBR system through a combination of practical experimental measurement and employing the previously published mathematical representation of the mechanical power demand $\bar{P^{\prime }}$ [5]. The outcomes are then compared with those reported for a pilot scale iHF MBR system also exclusively applying mechanical shear rather than air scouring of the membrane [8,9]. The values recorded can then be compared with SED values typical of full-scale iMBRs employing conventional membrane air scouring.

## 2. Materials and Methods

**Figure 2:**
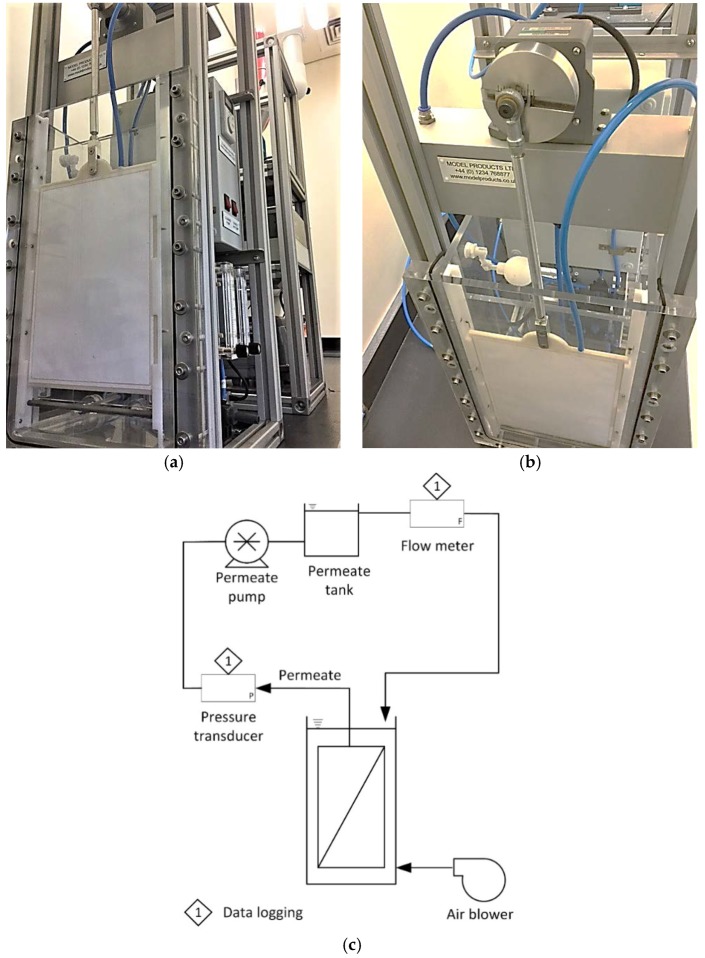
The membrane bioreactor (MBR) test cell, (**a**) membrane and channel; (**b**) motor and crank; and (**c**) schematic.

A single A4-sized 6 mm-thick FS panel (Kubota Membranes Europe, London, UK) was used to complete the testing, the complete test cell being based on what has recently been reported [10]. The cell (Figure 2a) comprised a ~500 mm-tall rectangular acrylic tank (130 × 245 mm) of around 20 L total volume. The membrane was placed in a conduit formed by a baffle between the tank and the outer cell wall, forming a 6 mm channel either side of the membrane. The membrane surface could then be viewed directly through the test cell wall, allowing sludging (i.e., the accumulation of solids filling the channel) to be measured by digital image analysis [10]. The non-visible side of the membrane was sealed so as to direct the permeate flow solely through the viewable side. The sludge in the process compartment was mixed using a fine-bubble aerator. Permeate was removed by suction with a peristaltic pump and fed to a 2 L de-aeration vessel prior to flowing through a digital flow meter, permeate pressure being also monitored using a digital pressure sensor (Figure 2c).

Reciprocation of the panel was through the fitting of a crank between the top of the panel and the arm of a motor (Figure 2b). This allowed the membrane to be reciprocated at a maximum rate of 20 RPM at an amplitude of 22 mm. The power demand in W per m^−2^ membrane area required for reciprocation was calculated according to the method of Buzatu et al. [5], based on a consideration of the vector forces acting on the membrane during the separate pushing and pulling contributions of the membrane reciprocation.

Tests were conducted on MBR sludge sampled from the membrane tank of a local 50 m^3^/day-capacity MBR wastewater treatment plant (WWTP) treating petroleum industry effluent. The sludge was characterized according to the standard sludge quality determinants of suspended solids (SS) concentration, particle size (using a Mastersizer 2000, Malvern Instruments Ltd., Malvern, UK), and capillary suction time (CST). All were measured according to standard methods [11].

Sludge samples were further characterized rheologically based on the measurement of viscosity over time using a controlled stress and strain rheometer (Anton Paar Model MCR 302, Graz, Austria) with a cup and bob configuration (DIN coaxial cylinder) at a temperature of 20 °C. Measurements were recorded following an equilibration time of 30 to 60 min at constant applied shear rate at fixed values of 20 to 120 s^−1^ for the sludge samples. Whereas the shear rate of 120 s^−1^ was considered to be representative of shear prevailing an immersed MBR [12], the mechanical shear applied in the study was considerably lower than this.

Filtration behavior was monitored according to the change in transmembrane pressure (TMP) and clogging propensity with time, the latter being recorded as the percentage coverage of the membrane with agglomerated solids (*%C*, in h^−1^) according to the method of Buzatu et al. [10]. Sludging was equated to the area of the membrane occluded by the sludge solids, as viewed through the transparent wall of the membrane test cell (Figure 2a). Images of the membrane were periodically captured and processed using an in-house Matlab program to determine the percentage of the membrane surface occluded. Surface coverage rates, expressed in % of coverage/h, were then calculated from the percentage occluded divided by the time passed from the start of the test.

Each test began at a sludge concentration of ~11 g·L^−1^ and filtration continued until the maximum recordable TMP of 0.6 bar was reached, the permeate being ejected such that the sludge solids concentration increased over the course of the test. The membrane was checked for clogging every 10 min and the time when the initiation of the clogging was observed recorded. The agglomerated solids were then dislodged at the end of the test and re-dispersed in the sludge. For all tests the sludge viscosity and filtrate COD (chemical oxygen demand) was recorded at the commencement of the test, and the sludge SS monitored as the solids concentration increased throughout the test.

Duplicated or triplicated measurements were taken for three different sludge samples. All tests were conducted at a flux of 15 L·m^−2^·h^−1^.

The calculation of the specific energy demand (SED) proceeded through:

- Determining the cycle-averaged shear rate *γ* in s^−1^ associated with the reciprocation rate of 20 RPM applied in the study;
- Practical measurement of the apparent viscosity *µ_a_* associated with this shear rate using the rheometer;
- Applying this viscosity to determine the specific power demand $\bar{P^{\prime }}$ W·m^−^^2^, based on the approach of Buzatu et al. [8], as outlined below;
- Calculating the SED, given by the ratio of $\bar{P^{\prime }}$ and the flux *J*.

The workings of Buzatu et al. [8] were used to calculate the mechanical power per unit membrane area (i.e., the specific power demand $\bar{P^{\prime }}$ W·m^−2^) associated with vertical reciprocation of the membrane in the MBR sludge suspension. This involved determining the force balance associated with the membrane motion, taking into account the buoyancy force of the membrane panel and the dynamic drag forces exerted on the membrane surface over the course of the cycle. The calculation encompassed the impact of shear on the apparent viscosity, the MBR sludge demonstrating shear-thinning behavior such that apparent viscosity decreases with the applied shear associated with reciprocation.

## 3. Results and Discussion

### 3.1. Practical Measurement

**Figure 3:**
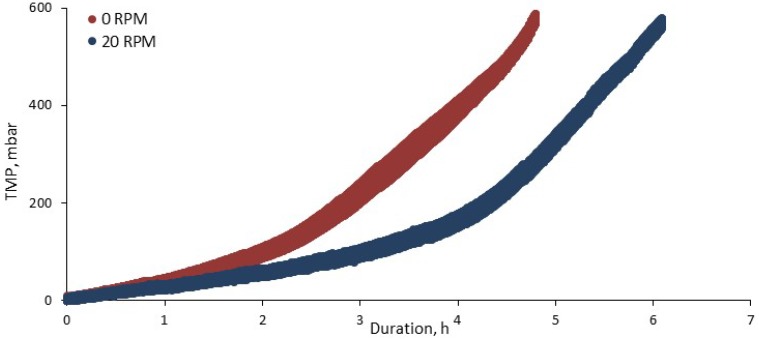
Example of impact of reciprocation on the pressure transient.

Data recorded across the study tended to indicate a small change in clogging and pressure incline rate as a result of application of the mechanical shear (Figure 3).

**Figure 4:**
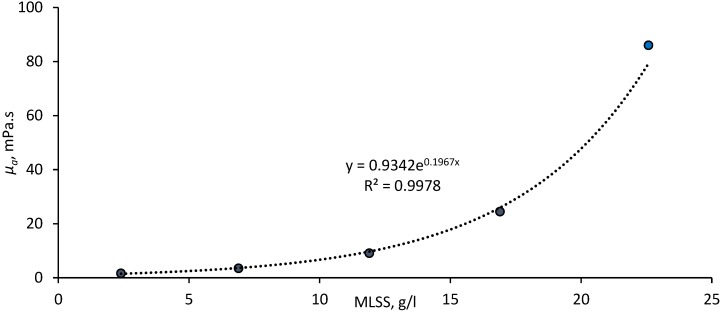
Viscosity as a function of solids concentration at a reference shear rate of 120 s^−1^.

**Figure 5:**
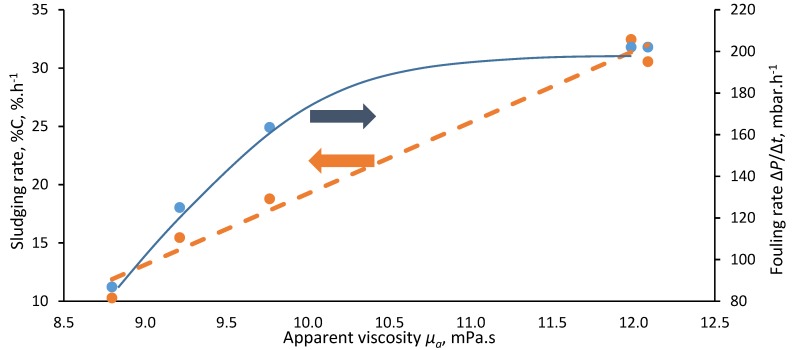
Sludging and fouling rates as function of apparent viscosity at a reference shear rate of 120 s^−1^.

Results revealed the viscosity of the sludge, as determined at a shear rate of 120 s^−1^ in accordance with the benchmark used previously [10], to follow an exponential relationship (Figure 4). This is in keeping with trends reported in a number of previous rheological studies [13,14,15]. Sludging (quantified as *%C*, the measured coverage or occlusion of the membrane surface by agglomerated solids per unit time) recorded in the absence of shear was revealed to increase with sludge viscosity (Figure 5), corroborating most recently reported work in this area [10]. Fouling, as notionally represented by Δ*P*/Δ*t*, was also found to increase with viscosity; reflecting the increased solids loading and greater hydraulic resistance imposed at the higher solids concentrations.

Sludging was also found to decrease, rather than increase, with dissolved COD across all measurements (11 data points between 60 and 100 mg·L^−1^, R^2^ = 0.84). This corroborates the previously reported observation of there being no link between fouling and sludging [10], since fouling would be expected to increase with soluble COD. In the current study the correlation between fouling and sCOD was weakly positive (R^2^ = 0.34).

**Figure 6:**
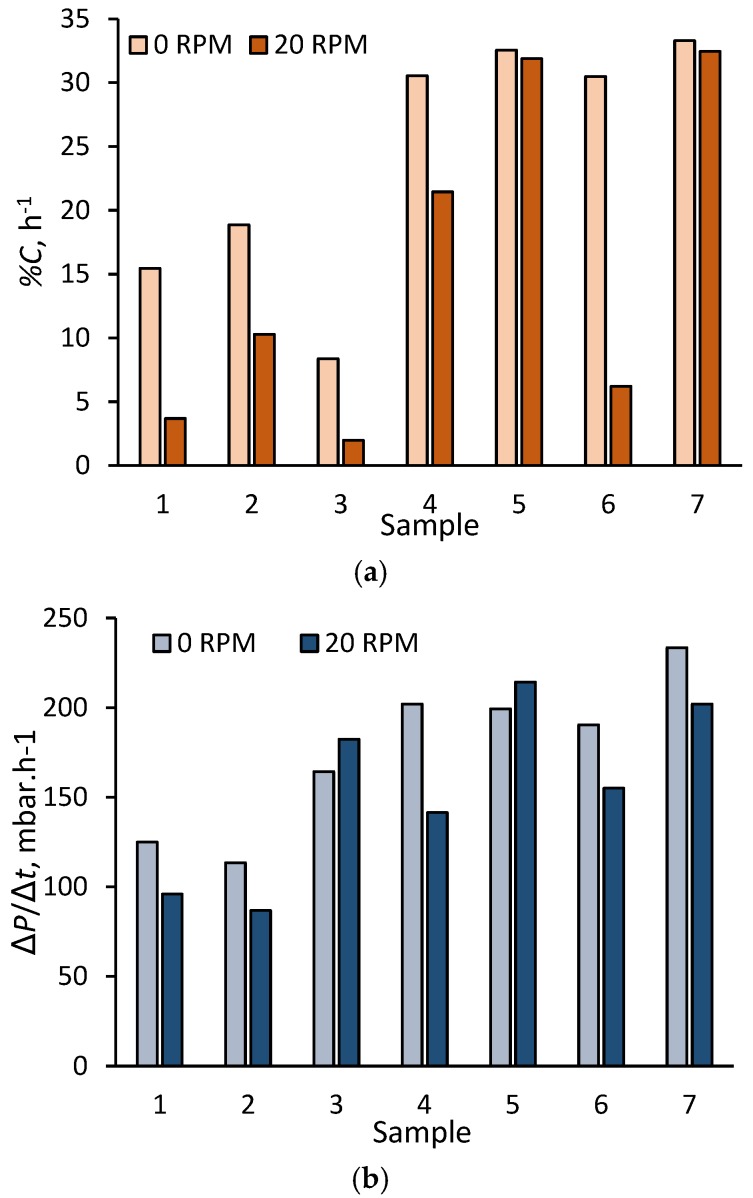
Rate of (**a**) percentage of membrane clogged and (**b**) pressure increase across seven samples, MLSS ~11 g·L^−1^, for stationary and reciprocating membrane (at 20 RPM).

A comparison of the impact of membrane reciprocation on the suppression of sludging (Figure 6a) and the pressure incline transient Δ*P*/Δ*t* (Figure 6b) revealed sludging to be more consistently affected than fouling. Across seven tests conducted on individual samples at an MLSS (mixed liquor suspended solids) concentration of around 11 g·L^−1^ (from 10.7 to 11.3 g·L^−1^), sludging decreased by between 2% and 80%, and by 45% on average over that measured for the stationary membrane. This compared with corresponding values of 11% to 30%, 13% on average, for the corresponding reduction in the Δ*P*/Δ*t* measurement.

A Student T-test analysis of the data for the clogging and fouling (Table 1) experimental data indicated the recorded suppression of these phenomena to be statistically significant. Both in the case of both the sludging and fouling rate data, the *P*(*T <= t*) *one-tail* value is larger than 0.05 and *t Stat* is lower than the *t Critical one-tail* value. This means that the null hypothesis (i.e., that the Hypothesized Mean Difference is zero) is rejected, i.e., that the means are significantly different. This then implies that the influence of membrane reciprocation on both clogging and fouling is statistically significant, according to this test method.

Outcomes suggest a small impact of applying a very small mean shear (4.9 s^−1^) on both the Δ*P*/Δ*t* and *%C* values, but with only the latter being of practical significance with regards to sustaining process operation. The size of the shear employed in the current study is to be compared with the considerably higher values of 2000 to 20,000 s^−1^ associated with rotating or vibrating disc systems [4], or the values 100 to 2000 s^−1^ employed in the original vibrating iHF studies [1]. For both these areas of study, shear has been applied as a means of increasing the flux through suppressing fouling, rather than clogging. Moreover, no consideration of the reasonable lower-limit the reciprocation rate has been given in these studies, whereas the calculations of Buzatu et al. [5] indicate that the energy benefit over classical air scouring increases with decreasing shear ate (below ~180 s^−1^) due to the difference in the value of the exponent in the power function for the specific power $\bar{P^{\prime }}$ with reference to the shear.

It is only the more recent pilot-scale studies [8,9] that have employed much lower reciprocation rates of 0.5 Hz or less, and consequently much reduced shear rates (Section 3.2), to produce significantly increased sustained flux values compared with classical air scouring [9]. As such, these authors’ data are germane to the current study, since similarly low shears were applied and beneficial impacts recorded (Figure 5) which was shown to be statistically significant (Table 1 and Table 2).

### 3.2. Determination of Energy Demand, Full-Scale Module

Applying the analytical method of Buzatu et al. [5], the cycle-averaged shear rate for the reciprocating membrane was calculated as being 0.24 times the rotation rate in RPM at the amplitude of 22 mm employed in the tests. For a full-scale module it is estimated that the amplitude would need to be increased to a maximum 100 mm to provide the same relative displacement. This yields a ratio of 1.1 between the cycle-averaged shear rate and the reciprocation frequency in RPM. Thus, at the reciprocation rate of 20 RPM applied in the study, the mean shear rate over the course of one cycle would be 22 s^−1^ (Table 2).

At this shear rate, according to the rheology measurements made, the apparent viscosity *µ_a_* of the sludge was measured as being 56 Pa·s on average. Employing the methodology of Buzatu et al. [5] the specific power demand $\bar{P^{\prime }}$ associated with this rotation speed and *µ_a_* value is 0.056 W·m^−2^, yielding an SED ($\bar{P^{\prime }}$/*J*) of 0.0081 kWh·m^−3^ (Table 2).

A comparison of the outcomes of the current study with those reported by Ho et al. [9] for a pilot-scale iHF MBR (Table 3) reveal a considerably lower SED for the current study, possibly due to the different calculational approaches taken and buoyancy assumptions made between the two studies. This is despite the more challenging nature of the sludge for the current study, taken from a full-scale operating iMBR treating petroleum industry effluent compared with the more benign municipal wastewater iHF MBR on which the Ho et al. study was based.

However, in both cases the SED is significantly lower than that associated with conventional air scouring of an immersed MBR. The most highly optimized classical coarse-bubble aerated system normally demands at least 0.15 kWh energy per m^3^ permeate [16,17,18], although membrane air-scour SEDs of ~0.08 kWh·m^−3^ have been claimed for the latest commercial technology [19], and lower still (around 0.04 kWh·m^−3^) for the most recently reported demonstration plants employing extensive pretreatment, and based on an extremely benign sludge [20].

## 4. Conclusions

The impact of a small-applied mechanical shear on the rate of pressure increase (Δ*P*/Δ*t*), normally assigned to fouling, and clogging (or, specifically, sludging—the agglomeration of sludge solids in the membrane channel) of a flat sheet (FS) membrane in an immersed membrane bioreactor (iMBR) has been assessed. Mixed liquor (or sludge) samples taken from a full-scale industrial effluent treatment MBR installation were used along with a dedicated test methodology. Sludging was quantified through visually determining the occlusion of the membrane surface by the attached solids.

The work has demonstrated:

- Small but statistically significant beneficial effects recorded from the application of the mechanical shear, manifested by reduced pressure incline and sludging rates, and
- The calculated energy demand associated with this mechanical shear application to be significantly lower than that recently reported for a pilot-scale demonstration.

Given the very low shear and related specific energy demand (0.008 kWh·m^−3^) to which this mechanical shear relates, further work would need to be conducted (a) at higher membrane reciprocation rates (i.e., higher shears); and/or (b) using combined mechanical shear and membrane air scour. The most frugal commercially-available membrane air-scour system operates at an air-scour SED of ~0.08 kWh·m^−3^, around ten times greater than the equivalent SED used in the current study. Higher mechanical shear rates would provide lower apparent viscosities due to the sludge rheological characteristics, compensating for the increased power consumption associated with the higher motor rotation rates.

However, the outcome of most practical significance from the current study is the tangible impact on sludging amelioration. Whilst it is unlikely that mechanically-applied shear would be practical on large-scale systems, the ability to de-sludge a clogged membrane through applying low-energy reciprocation to a small number of modules may prove be economically viable due to the considerable savings in labor and downtime expenditure. It is also the case that the dynamics of the shear over the course of a complete cycle can be adjusted simply by altering the cam or rotor geometry. Finally, the energy demand of mechanical shear is sufficiently low as to permit the possibility of combined coarse-bubble aeration and mechanical shear, which may prove as energetically-efficient, but more robust than classical aeration alone. There is thus clearly much left to explore in the areas of mechanical shear and membrane sludging amelioration in the further development of MBR technology.

## Figures and Tables

**Table 1:** Clogging data, Student T Test analysis.

Parameter	Sludging Rate Data		Fouling Rate Data	
	20 RPM	0 RPM	20 RPM	0 RPM
Mean	12.6	22.1	146.0	165.4
Variance	138	84.2	2418	1243
Observations	6	7	6	7
Pooled variance	109		1777	
Hypothesized mean difference	0		0	
Degrees of freedom	11		11	
*t Stat*	−1.65		−0.829	
*P(T <= t) one-tail*	0.063		0.212	
*t Critical one-tail*	1.80		1.80	
*P(T <= t) two-tail*	0.127		0.425	
*t Critical two-tail*	2.20		2.20	

**Table 2:** Calculated parameter values, according to the approach of Buzatu et al. [5].

Parameter	Units	Bench-Scale	Full-Scale
Frequency	RPM	20	20
*γ*	s^−1^	4.9	22.2
Amplitude	m	0.022	0.1
*η_a_*	mPa·s	153	56.4
*P*	W·m^−2^	0.00839	0.07263
*J*	LMH	15	15
Motor efficiency	%	60%	60%
SED	kWh·m^−3^	0.00093	0.00807

**Table 3:** Results from current study vs. published pilot-scale mechanical shear, Ho et al. [8,9].

Parameter	Unit	Ho et al. [8,9]	This Study
*Design and operation*			
Membrane area	m^2^	45	0.1
Membrane length	m	1.3	0.316
TMP	kPa	<20	<60
Amplitude	mm	38 to 57	100
Reciprocation frequency	Hz	0.38 to 0.53	0.33
	RPM	23 to 32	20
Specific power demand $\bar{P^{\prime }}$	W·m^−2^	1.55	0.073 ^a^
*Experimental outputs*			
Flux (*J*) range	L·m^−2^·h^−1^ or LMH	20 to 40	15
Permeability	LMH·bar^−1^	200 to 300	25 to 180
SED ($\bar{P^{\prime }}$/*J*)	kWh·m^−3^	0.04 to 0.09	0.0081 ^b^
Motor efficiency	-	71%	60%

^a^ Determined according to the method of Buzatu et al. [5]. ^b^ Based on mechanical energy demand only.
